# Graphene Enhanced Secondary Ion Mass Spectrometry (GESIMS)

**DOI:** 10.1038/s41598-017-07984-1

**Published:** 2017-08-07

**Authors:** Paweł Piotr Michałowski, Wawrzyniec Kaszub, Iwona Pasternak, Włodek Strupiński

**Affiliations:** Institute of Electronic Materials Technology, Wólczyńska 133, 01-919 Warsaw, Poland

## Abstract

The following invention - Graphene Enhanced Secondary Ion Mass Spectrometry - (pending European patent application no. EP 16461554.4) is related to a method of analysing a solid substrate by means of Secondary Ion Mass Spectrometry (SIMS). It comprises the steps of providing a graphene layer over the substrate surface and analysing ejected secondary anions through mass spectrometry analysis. The graphene layer acts as a kind of filament that emits a lot of secondary electrons during the experiment which significantly increases the negative ionization probability and thus the intensity of the SIMS signal can be more than two orders of magnitude higher than that of a similar sample without graphene. The method is particularly useful for the analysis of surfaces, 2D materials and ultra-thin films. The intensity of dopants and contamination signals can be enhanced up to 35 times, which approaches the detection limit of ~10^15^ *atoms*/*cm* ^3^, otherwise unreachable in a standard static SIMS analysis.

## Introduction

SIMS is a very precise analytical technique for determining the elemental composition of a sample^1–6^. The sample is bombarded with a primary ion beam that leads to the sputtering of the surface. Small parts of the sputtered particles are ionized (secondary ions) and the sample composition can be determined by means of mass spectral analysis. During the sputtering subsequent layers of the sample are removed and thus it is possible to obtain information about changes in the composition as a function of depth, thus creating a depth profile.

SIMS is well known for its excellent detection limits of trace elements^7–12^. For most materials it is reported to be in the range of 10^15^–10^16^ *atoms*/*cm* ^3^ ^11^, sometimes even as good as 10^12^ *atoms*/*cm* ^3^ ^12^. These optimum detection limits, however, are achieved during the dynamic SIMS (dSIMS) mode. In this mode a very dense ion beam is used and a substantial amount of material is sputtered simultaneously, allowing more ions to be detected. Drawbacks include very poor depth resolution and limitation to thick materials.

Surfaces, 2D materials and ultra-thin films are analysed ideally in a special mode called static SIMS (sSIMS)^13–15^, in which a low density ion beam ensures that ions are emitted only from monolayers 1 to 3. This mode, however, has physical limitations. Because a relatively small amount of matter is sputtered, and even less ionized, there are not enough secondary ions extracted from the sample to achieve as optimum detection limit as the dynamic mode does. In most cases it is difficult to exceed a limit of 1 ppm (5 * 10^16^ *atoms*/*cm* ^3^ or 10^9^ *atoms*/*cm* ^2^).

There are many ways to enhance the ionization probability and thus the detection limit. It has been noted that using oxygen as primary ions significantly enhances the formation of positive ions^16–19^, whereas cesium enhances the formation of negative ions^17–20^. For some elements the difference can be up to four orders of magnitude. Although most elements can be detected as both positive and negative ions, each element usually gives better results in terms of detection limit in either positive or negative mode. In some experiments, oxygen flooding can further enhance the secondary ion yields of some negative ions^21–23^. Using these techniques yields acceptable results, but in the cases of surfaces, 2D materials and ultra-thin films, it is often not enough to reach the desired detection limit, particularly in the case of trace elements.

Several SIMS measurements on graphene (Gr) have already been reported^24–31^. More specifically, SIMS has been used to map the surface of the graphene layer^24, 25^, to analyse the mass spectra of graphene^26, 27^, and to create a depth profile of single-layer graphene grown on substrates that do not contain carbon^28, 29^. Furthermore, SIMS has been used for the depth profiling of multilayer graphene of a thickness of more than 3 nm on SiC^30^ and, recently, of hydrogen-intercalated graphene on SiC having a theoretical thickness of 0.69 nm^31^. All these methods employed SIMS for characterising the graphene layer, while the composition of support material underneath was not analysed.

In this work we present Graphene Enhanced Secondary Ion Mass Spectrometry (GESIMS) - a new technique for enhancing the detection limit in the analysis of thin materials. It has been found that providing a graphene layer on a substrate significantly reduces the emission of matter from the substrate but at the same time increases the negative ionization probability. Destruction of a small part of the graphene layer in a dynamic SIMS mode significantly reduces the initial strong blocking effect of graphene, while the enhancement of the ionization probability of the sputtered material is still observed. Under these conditions the ionization probability greatly prevails, resulting in an SIMS signal with an intensity that is unexpectedly high compared with a sample without graphene and thus able to to reach better detection limits.

## Results

**Figure 1:**
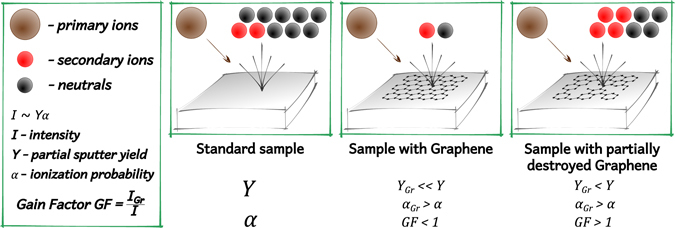
The basic concept of GESIMS analysis. Graphene blocks the emission of the matter from the substrate but significantly increases the ionization probability. By creating some defects in graphene layer the emission increases while the ionization ability is preserved and thus more ions can reach the detector, resulting in enhanced SIMS signal.

**Figure 2:**
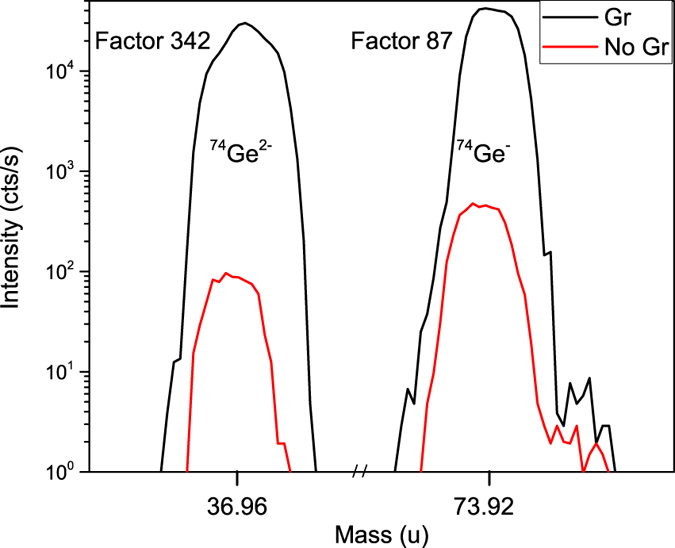
A comparison of mass spectra of the germanium substrate with and without graphene layer for optimal GESIMS conditions. The intensity of the SIMS signal can be enhanced more than two orders of magnitude.

The basic concept of GESIMS method is presented on Fig. 1. To understand the process correctly it is necessary to remember that the intensity of SIMS signals depends on the secondary ion yield, which is defined as the number of emitted ions *A* ^−^/*A* ^+^ (multiple ionization is also possible) per incident ion. In turn, secondary ion yield is defined as the product of the partial sputter yield (number of emitted species A per incident ion) and ionization probability. The presence of graphene on a substrate surface clearly reduces the partial sputter yield of a material from the substrate, since for given conditions it is expected that sputtered species are emitted from the ~3 top monolayers of material and graphene acts as a barrier to emission. If despite this fact a significant increase in the SIMS signal intensity is still observed, the only possible explanation is that the presence of graphene drastically increases the negative ionization probability. Fig. 2 presents a comparison of mass spectra for the Ge substrate with and without a graphene layer for optimal GESIMS conditions (the graphene layer was partially destroyed prior to the analysis). From this, it is possible to conclude that graphene can enhance the intensity of peaks by two orders of magnitude.

To quantify the effect, a gain factor can be defined as follows:

1 $$GF=\frac{{Y}_{Gr}{\alpha }_{Gr}}{Y\alpha }$$

wherein GF is the gain factor, *Y*_*Gr*_ and *Y* denote partial sputter yield for a sample with and without graphene, respectively, while *α*_*Gr*_ and *α* denote ionization probabilities (again, a sample with and without graphene, respectively). Under optimum conditions *Y*_*Gr*_ < *Y* and *α*_*Gr*_ > *α*, whereby the latter effect is much stronger, so that *GF* > 1 and thus the intensity of the SIMS signal can be enhanced.

Although increasing the intensity of the matrix element signal is of interest from a scientific viewpoint, it does not seem to have much practical application in SIMS-based surface analysis. As already mentioned above, the actual benefit is the enhancement of the detection limit of trace elements and thus new sets of samples have been considered and tested. Each of the sets consisted of a pair of samples, one having graphene transferred onto its surface, and the other - without graphene - serving as a reference. Signal enhancement was observed for all of them and the maximum gain that was reached during the experiments has been presented in Table 1 along with standard and enhanced detection limits.

**Table 1:** Summary of Graphene Enhanced Secondary Ion Mass Spectrometry measurements performed on various materials and dopants.

Substrate	Dopant	Concentration (*atoms*/*cm* ^3^)	Maximal gain factor	Standard detection limit (*atoms*/*cm* ^3^)	Enhanced detection limit (*atoms*/*cm* ^3^)
GaAs	Te	1 * 10^18^	35.7	7.04 * 10^16^	1.97 * 10^15^
Si	Sb	1 * 10^18^	15.1	4.86 * 10^16^	3.02 * 10^15^
AlGaAs	Si	2 * 10^18^	13.8	6.06 * 10^16^	4.39 * 10^15^
Si	As	5 * 10^18^	7.1	1.35 * 10^16^	1.90 * 10^15^

**Figure 3:**
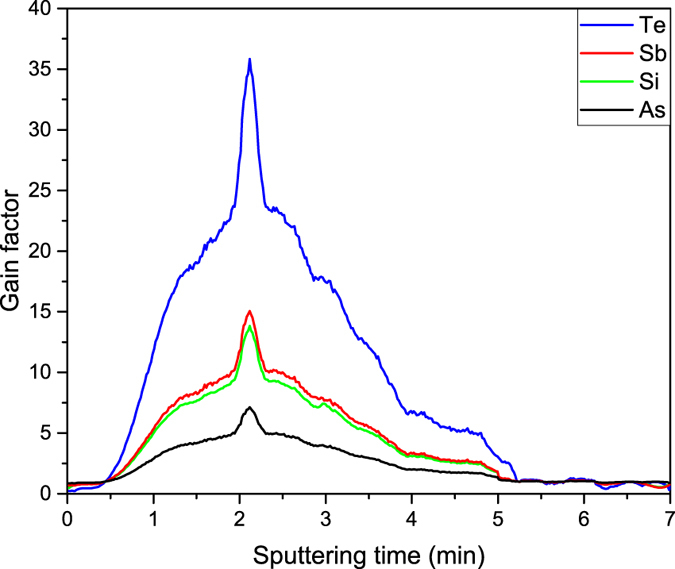
The gain change during sputtering in dSIMS mode for different dopant types in various substrates. At the beginning the blocking effect is very strong but after the partial destruction of graphene it is reduced and the enhanced ionization probability is dominant. Thus the gain is much larger than a factor of 1. After the complete destruction of the graphene layer, no difference between the two samples is observed (*GF* = 1). The shape of all curves and the position of the maximum depends on the partial destruction of the graphene layer and thus are similar for all samples.

As already mentioned, the effect was not observed at the beginning of the experiment, when a fresh graphene layer was transferred on a sample. This is not surprising since graphene is a strong material and the blocking effect was expected to be high. Thus the reduction in the partial sputter yield was higher than the increase of the ionization probability. To overcome it, the primary beam intensity had to be increased to several hundred pA for a short time. Under such conditions dynamic SIMS mode was achieved and the entire surface was slowly eroded. When only a small part of the graphene sheet was destroyed, the blocking effect was significantly reduced while the enhancement of the ionization probability was still observed. Figure 3 shows the gain change during sputtering in dSIMS mode with the primary beam intensity being set to 200 pA for all samples presented in Table 1. Three regions can be identified: at the beginning of the experiment the blocking effect was too strong and thus the signal for the sample with graphene was reduced (GF < 1). After sputtering off a small part of the graphene, the enhanced ionization probability took the major role and thus the gain was much larger than a factor of 1 (GF ≫ 1). If the beam intensity was kept at high values for a longer time, complete destruction of the graphene layer took place, and consequently no difference between the two samples in each set was further observed (*GF* = 1).

It was essential in these experiments for the uniformity and the intensity of the beam to be well defined and reproducible and thus it was not surprising that the shape of all curves and the position of the maximum were similar for all measured samples as they depended on the partial destruction of the graphene layer and not to the type of the dopant and the substrate. Only the actual enhancement was material related (both the substrate and the type of dopant) and thus each sample had a different maximal gain factor.

**Figure 4:**
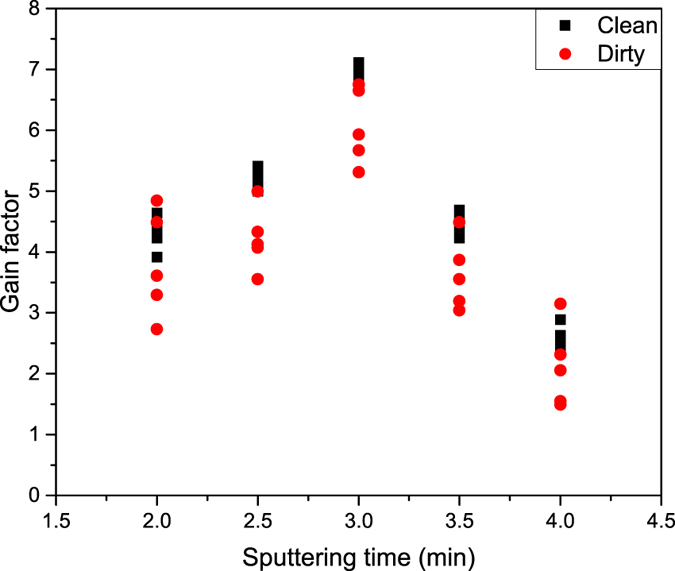
The gain change during sputtering in dSIMS mode for As dopant in Si substrate measured at five different spots for a clean and a dirty sample. For the cleaned sample, the method is reproducible, with small variations only.

The result is fully reproducible - Fig. 3 shows the average of 15 measurements performed separately on both types of sample (with and without graphene) for each sample set. We wish to emphasize that to achieve a good reproducibility of the method, samples must be cleaned prior to the measurement by annealing them at elevated temperatures and reduced pressure. In the experiment described above, the samples were subjected to annealing at 200 °C and pressure of 10^−8^ *mbar* for two hours. Figure 4 shows a suitable comparison of the gain factors measured for As dopant in Si substrate samples with and without cleaning procedures. The intensity of the primary beam was set to 150 pA and the duration of the dSIMS mode was varied between 2 and 4 minutes. Five different measurements were taken at various spots of the sample, leading to a straightforward conclusion that for the cleaned sample the method is reproducible, with only small variations.

**Figure 5:**
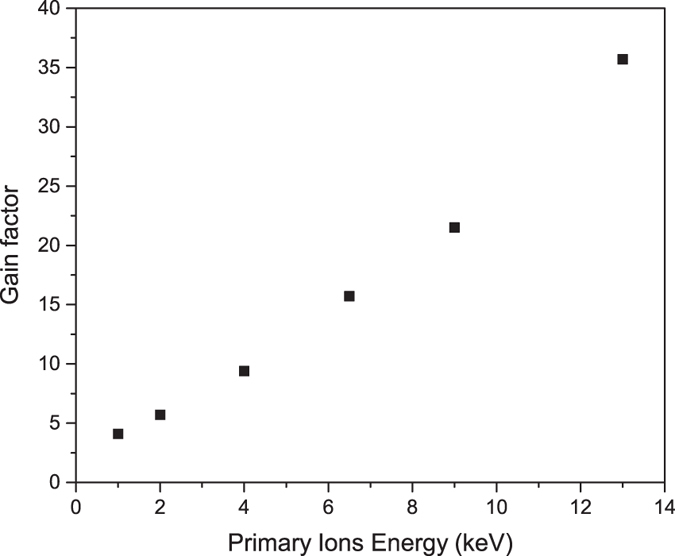
Maximal gain factor as a function of primary ion energy for Te dopant in GaAs substrate. For high-energy ions, a bigger collision cascade is generated and thus it is easier to overcome the graphene blocking effect.

The best results, as presented in Table 1, were obtained for higher values of primary ion energy (usually above 10 keV). This is not surprising because as more energy is transferred to the substrate, a larger collision cascade is generated and thus the matter from the substrate has higher energy to overcome the graphene blocking effect. Figure 5 shows a comparison of maximal gain factor versus primary ion energy for Te dopant in GaAs substrate.

**Figure 6:**
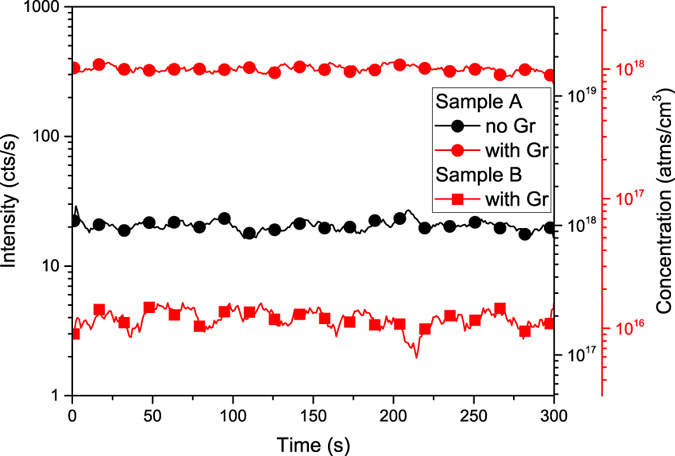
Static SIMS mode measurement for Sb dopant in Si substrate for samples with various dopant concentration: 1 * 10^18^ and 1 * 10^16^ *atoms*/*cm* ^3^ for Sample A and B, respectively. The presence of the graphene layer significantly increases the intensity of the SIMS signal, improving the detection limit. The GESIMS method allows the analysis of samples with low concentration, which is not possible using the standard measurement.

When the optimal conditions are reached, the beam intensity should be decreased and sSIMS measurement can be performed. Figure 6 presents a suitable comparison of SIMS measurement for Sb dopant in Si substrate with and without graphene. Two sample sets were analysed: with high (1 * 10^18^ *atoms*/*cm* ^3^) and low (1 * 10^16^ *atoms*/*cm* ^3^) dopant concentration (Sample A and B, respectively). As can be clearly seen for Sample A, the intensity of the SIMS signal was significantly increased and thus a better detection limit was achieved (3.02 * 10^15^ instead of 4.86 * 10^16^ *atoms*/*cm* ^3^). In the case of Sample B, the concentration was too low to be measured using a standard sSIMS condition, whereas it was possible for the GESIMS method. As can be seen, the enhancing effect does not depend on the dopant concentration and thus GESIMS method is quantifiable.

**Figure 7:**
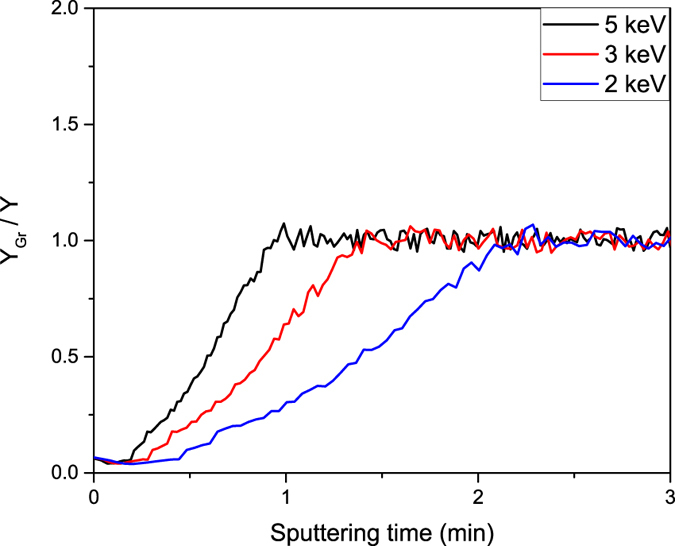
The gain change during sputtering in dSIMS mode for Ga^+^ ions in GaAs substrate. In positive ion detection, no enhancement effect is observed, only the blocking effect of graphene. The positive ionization probability remains the same for the whole experiment and thus the *Y*_*Gr*_/*Y* ratio is being monitored. For smaller impact energies lower sputter rate is observed.

The same procedure was used for a O_2_^+^ primary beam with positive ion detection. As shown on Fig. 7 only the blocking effect of graphene was observed with no enhancement. Changing the impact energy influenced the sputter rate whereas the general shape of the profile was the same. It was therefore concluded that the positive ionization probability remained the same for the whole experiment and only the sputter yield increased while the graphene layer was being destroyed, therefore the oxygen beam was found suitable to determine the *Y*_*Gr*_/*Y* ratio.

**Figure 8:**
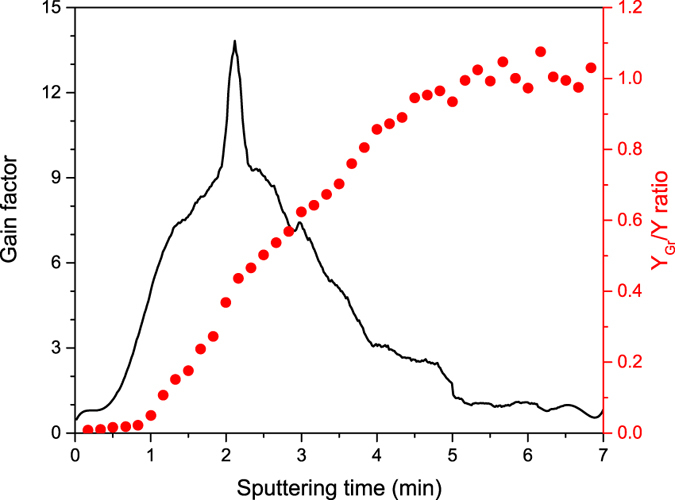
A comparison of the gain change during sputtering in dSIMS mode and the *Y*_*Gr*_/*Y* ratio for Si dopant in AlGaAs substrate. Close to the optimal condition the *Y*_*Gr*_/*Y* ratio increases significantly whereas the enhanced ionization probability is still present.

A new experiment was devised for AlGaAs substrate with Si dopant: the sample was sputtered in dSIMS mode with Cs^+^ ions and the gain factor change was obtained just like it was shown on Fig. 3. Every ten seconds the Cs^+^ beam was switched off and a very brief sSIMS measurement with O_2_^+^ beam was performed so that the *Y*_*Gr*_/*Y* ratio could be determined as shown on Fig. 8. We noted that the *Y*_*Gr*_/*Y* ratio increased rapidly from ~0.27 to ~0.43 close to the optimal condition which apparently led to a significant increase of the gain factor.

It did not, however, explain why the graphene layer increase the negative ionization probability. To better understand the effect we repeated the whole procedure but instead of the graphene layer we deposited a thin layer of amorphous carbon. We tested it in both, negative and positive ion detection mode but no signal enhancement was observed, just the blocking effect and thus the enhancement could not be associated with the presence of carbon atoms only. To gain more insight we studied the electric properties of the graphene layer and amorphous carbon during the SIMS experiment: we transferred/deposited them on insulating materials like SiC, AlN and SiO_2_ which normally required an electron flood gun for charge compensation. The intensity of the Cs^+^ primary beam was once again set to 200 pA and it turned out that a thin layer of the amorphous carbon did not alter the experiment significantly: the charging effect was visible from the very beginning and the flood gun was needed to perform the analysis. However, in case of the graphene layer we were able to sputter the sample without the flood gun up to four an a half minutes before the charging effect occurred. It corresponded well with the time frame of the enhancement effect (see Fig. 3).

From Fig. 8 it could be further deduced that after four and half minutes of sputtering the graphene layer was significantly destroyed (the *Y*_*Gr*_/*Y* ratio was above 0.8) and yet it was still able to provide enough electrons for charge compensation. We assumed that in a similar way the GESIMS effect could be explained: during the SIMS experiment a high voltage was applied to the sample holder (up to 5 kV) and thus the graphene layer acted as a kind of filament which emitted an excess of electrons during the ion bombardment leading to the enhanced ionization probability.

## Discussion

GESIMS is a process for measuring and analysing thin substrates that involves the following steps:

- applying a graphene layer over the substrate surface;
- annealing the graphene-coated substrate at elevated temperature and reduced pressure;
- sputtering of the graphene-coated substrate in dSIMS mode, which leads to the partial destruction of the graphene layer;
- detecting and analysing ejected secondary anions by mass spectrometry analysis in sSIMS mode.

This method cannot be used for depth profiling of thick materials because the graphene layer will be destroyed within the first moments of the measurement and the enhancing property will be lost. Furthermore, the dSIMS analysis itself offers a high detection limit. It cannot be used for a high resolution imagining neither, since the presence of the partially destroyed graphene layer will reduce the spatial resolution. However, in the case of very thin materials, sSIMS reaches its physical limit and any enhancement is beneficial. GESIMS requires more preparation and optimization than the standard sSIMS analysis but improves by one order of magnitude the detection limits of trace elements, which may prove to be invaluable for the further development of 2D materials.

The effect does not take place for samples covered with a thin layer of amorphous carbon. It has been noted that the graphene layer has different electric properties during the SIMS experiment - even a significantly destroyed layer can provide enough electrons to compensate the charging effect which is typically present for insulating materials. This behaviour can suggest the explanation of the GESIMS effect: since a high voltage is applied to the sample holder the graphene layer acts as a kind of filament and emits a lot of secondary electrons during the ion bombardment and thus the negative ionization probability is increased.

## Methods

### Sample preparation

A pair of samples was created for every experiment described in this work, one having graphene grown or transferred onto its surface, and the other - without graphene - serving as a reference.

In the case of germanium substrate, graphene films were synthesized in a commercially available system by the CVD method as described by Paternak *et al*.^32, 33^. As a substrate, (100)-oriented Ge layers deposited on Si (100) wafers were used. Methane gas in the mixture of Ar and *H*_2_ in the ratio of 200:1 was used as a carbon precursor. Growth was preceded by the substrate’s annealing in a pure hydrogen atmosphere in order to reduce native oxides *in-situ*. During the growth process, 800 mbar of pressure were sustained.

In other samples, graphene films were synthesized on 35 *μm* thick copper foils by the CVD method using a Black Magic Pro system (Aixtron). To grow graphene on copper foils the following procedure was used: copper samples were first pretreated at 960 °C under an Ar gas flow and then a *H*_2_ gas flow at 20 mbar of pressure. The purpose of this step was to improve the quality and enlarge the grain size of Cu substrates as well as to remove oxides from the copper surface. Next, methane was introduced into the reactor at an adequate flow rate for a few minutes. During this step, the synthesis of graphene was observed. Finally, the copper substrates covering the graphene films were cooled to room temperature in an Ar atmosphere. To transfer graphene onto target substrates, we used the high-speed electrochemical delamination method^34^.

As described in aforementioned procedures, the presence of the graphene layer was confirmed by Raman spectroscopy measurements performed in back-scattering geometry, using a Renishaw inVia Raman Microscope, with a ×100 objective and a 532 nm Nd:YAG laser as an excitation source.

### SIMS measurements

In this work all SIMS measurements were performed employing the CAMECA SC Ultra instrument under ultra-high vacuum (UHV), usually of 4 × 10^−10^ *mbar*. A Cs^+^ primary beam with negative ion detection was used for most measurements. Impact energy was varied between 1 keV and 13 keV but the highest energy was used for the most of the experiments. The intensity of the primary beam was 100–2000 pA and 0.5–10 pA for the dynamic and static SIMS mode, respectively. An O_2_^+^ primary beam with positive ion detection was used for a few experiments to show that graphene do not enhance the positive ionization probability. Impact energy was varied between 2 keV and 5 keV and the intensity of the primary beam was 300–3000 pA and 3–10 pA for the dynamic and static SIMS mode, respectively. Primary beams were rastered over 250 × 250 *μm* ^2^ and the analysis area was limited to (200 × 200) *μm* ^2^. Mass interferences were verified prior to analysis and adequate mass resolving power was used for each experiment.

Beam on the sample in the SC Ultra tool has a square shape and owning to the “variable rectangular shape concept” forms a homogeneous spot. The primary beam at the working point in the SC Ultra is formed by two stencils - well-shaped apertures. While the first one is used to choose the most intense and homogeneous part of the ion beam, the second one changes the size of the spot. This innovation provides high sensitivity for all measured elements and a high dynamic range with a very low sputter rate^35–37^.

Secondary ions that are detected in SIMS analysis usually originate from the top three monolayers of the bombarded sample. For static SIMS analysis, very low ion doses are used (typically in range of 10^12^ *ions*/*cm* ^2^). This ensures that with a high probability every ion will impact on undamaged surface and not on an area that has already received ion impacts. In this mode, the emission is essentially limited to a few topmost monolayers and thus it is very surface-sensitive. It makes it an ideal mode for the analysis of surfaces, 2D materials and ultra-thin films because there is no interference with the lower layers. In dynamic SIMS mode the emission is theoretically still limited to the topmost monolayers but for higher ion doses it is expected that the whole surface will erode in time and thus the lower layer will be gradually exposed. In this way it is possible to obtain information on the variation of the composition of material below the initial surface, thereby creating so-called depth profiles. In this work, however, the dynamic SIMS mode was used to destroy only partially the graphene layer in order to expose the underlying substrate, which was subjected for further analysis. It was a critical step to ensure good reproducibility of the results and therefore a very homogeneous ion beam was required to perform GESIMS analysis.

## References

[1] Andersen CA, Hinthorne JR (1972). Ion microprobe mass analyzer. Science.

[2] Benninghoven A (1975). Developments in secondary ion mass spectroscopy and applications to surface studies. Surf. Sci..

[3] Benninghoven, A., Rudenauer, F. G. & Werner, H. W. Secondary ion mass spectrometry: basic concepts, instrumental aspects, applications and trends (John Wiley & Sons, New York, 1987).

[4] Werner HW (1975). The use of secondary ion mass spectrometry in surface analysis. Surf. Sci..

[5] Liebl H (1975). Secondary-ion mass spectrometry and its use in depth profiling. J. Vac. Sci. Technol., A.

[6] Liebl H (1967). Ion microprobe mass analyzer. J. Appl. Phys..

[7] Wittmaack K (1976). High-sensitivity depth profiling of arsenic and phosphorus in silicon by means of sims. Appl. Phys. Lett..

[8] Ber BY (1998). Secondary ion mass spectroscopy investigations of magnesium and carbon doped gallium nitride films grown by molecular beam epitaxy. Semicond. Sci. Technol..

[9] Chiou CY, Wang CC, Ling YC, Chiang CI (2003). Secondary ion mass spectrometry analysis of in-doped p-type gan films. Appl. Surf. Sci..

[10] Emziane M, Durose K, Halliday DP, Bosio A, Romeo N (2006). *In situ* oxygen incorporation and related issues in cdte/cds photovoltaic devices. J. Appl. Phys..

[11] Matsunaga T, Yoshikawa S, Tsukamoto K (2002). Secondary ion yields of c, si, ge and cs surface density and concentration in sims. Surf. Sci..

[12] Gnaser H (1997). Sims detection in the 10^12^ atoms *cm*^−3^ range. Surf. Interface Anal..

[13] Benninghoven A (1994). Chemical analysis of inorganic and organic surfaces and thin films by static time-of-flight secondary ion mass spectrometry (tof-sims). Angew. Chem. Int. Ed..

[14] VanVaeck L, Adriaens A, Gijbels R (1999). Static secondary ion mass spectrometry: (s-sims) part 1. methodology and structural interpretation. Mass Spectrom. Rev..

[15] Adriaens A, VanVaeck L, Adams F (1999). Static secondary ion mass spectrometry (s-sims) part 2: Material science applications. Mass Spectrom. Rev..

[16] Novak SW, Wilson RG (1991). Systematics of positive secondary ion mass spectrometry relative sensitivity factors for si and sio_2_ measured using oxygen and argon ion bombardment. J. Appl. Phys..

[17] Slodzian G, Lorin JC, Havette A (1980). Isotopic effect on the ionization probabilities in secondary ion emission. J. Phys. Lett. Paris.

[18] Taga Y (1986). Sputtering and secondary ion emission from metals and alloys subjected to oxygen ion bombardment. Secondary Ion Mass Spectrometry SIMS V.

[19] Gnaser H, Hutcheon ID (1988). Significance of isotope effects for secondary-ion emission models. Phys. Rev. B.

[20] Stevie FA, Kahora PM, Simons DS, Chi P (1988). Secondary ion yield changes in si and gaas due to topography changes during o+2 or cs+ ion bombardment. J. Vac. Sci. Technol., A.

[21] Lewis RK, Morabito JM, Tsa JC (1973). Primary oxygen ion implantation effects on depth profiles by secondary ion emission mass spectrometry. Appl. Phys. Lett..

[22] Vickerman, J. C., Brown, A. & Reed, N. M. *Secondary ion mass spectrometry: Principles and applications* (Clarendon Press, Oxford, 1989).

[23] Wilson, R. G., Stevie, F. A. & Magee, C. W. Secondary ion mass spectrometry: A practical handbook for depth profiling and bulk impurity analysis (John Wiley & Sons Inc., New York, 1989).

[24] Luo Z (2011). Pyridinic n doped graphene: synthesis, electronic structure, and electrocatalytic property. J. Mater. Chem..

[25] Lupina G (2015). Residual metallic contamination of transferred chemical vapor deposited graphene. ACS Nano.

[26] Dwivedi N (2015). Ultrathin carbon with interspersed graphene/fullerene-like nanostructures: A durable protective overcoat for high density magnetic storage. Sci. Rep..

[27] Xie W, Weng L-T, Ng K, Chan C, Chan C-M (2015). Clean graphene surface through high temperature annealing. Carbon.

[28] Li Q (2013). Growth of adlayer graphene on cu studied by carbon isotope labeling. Nano Lett..

[29] Chou H, Ismach A, Ghosh R, Ruoff R, Dolocan A (2015). Revealing the planar chemistry of two-dimensional heterostructures at the atomic level. Nat. Commun..

[30] Yannopoulos S (2012). Co2-laser-induced growth of epitaxial graphene on 6h-sic(0001). Adv. Funct. Mater..

[31] Michalowski PP, Kaszub W, Merkulov A, Strupinski W (2016). Secondary ion mass spectroscopy depth profiling of hydrogen-intercalated graphene on sic. Appl. Phys. Lett..

[32] Pasternak, I. *et al*. Graphene growth on ge(100)/si(100) substrates by cvd method. *Sci. Rep*. **6**, 21773 (2016). 10.1038/srep21773 PMC4761869 26899732

[33] Pasternak I (2016). Large-area high-quality graphene on ge(001)/si(001) substrates. Nanoscale.

[34] Ciuk T (2013). Properties of chemical vapor deposition graphene transferred by high-speed electrochemical delamination. J. Phys. Chem. C.

[35] CAMECA, Genneviliers. *CAMECA SC-Ultra, User’s Guide* (2005).

[36] Kouzminov D, Merkulov A, Arevalo E, Grossmann. HJ (2013). Application of extra-low impact energy sims and data reduction algorithm to usj profiling. Surf. Interface Anal..

[37] Merkulov A (2013). The secondary ions emission from si under low-energy cs bombardment in a presence of oxygen. Surf. Interface Anal..

